# A DNA barcode reference library of French Polynesian shore fishes

**DOI:** 10.1038/s41597-019-0123-5

**Published:** 2019-07-04

**Authors:** Erwan Delrieu-Trottin, Jeffrey T. Williams, Diane Pitassy, Amy Driskell, Nicolas Hubert, Jérémie Viviani, Thomas H. Cribb, Benoit Espiau, René Galzin, Michel Kulbicki, Thierry Lison de Loma, Christopher Meyer, Johann Mourier, Gérard Mou-Tham, Valeriano Parravicini, Patrick Plantard, Pierre Sasal, Gilles Siu, Nathalie Tolou, Michel Veuille, Lee Weigt, Serge Planes

**Affiliations:** 1 Institut de Recherche pour le Développement, UMR 226 ISEM (UM2-CNRS-IRD-EPHE), Université de Montpellier, Place Eugène Bataillon, CC 065, F-34095 Montpellier, cedex 05 France; 2 Museum für Naturkunde, Leibniz-Institut für Evolutions-und Biodiversitätsforschung an der Humboldt-Universität zu Berlin, Invalidenstrasse 43, Berlin, 10115 Germany; 3 PSL Research University, EPHE-UPVD-CNRS, USR 3278 CRIOBE, Université de Perpignan, 58 Avenue Paul Alduy, 66860 Perpignan, France; 4 Laboratoire d’Excellence «CORAIL», Papetoai, Moorea, French Polynesia France; 5 Division of Fishes, Department of Vertebrate Zoology, National Museum of Natural History, Smithsonian Institution, 4210 Silver Hill Road, Suitland, MD 20746 USA; 6 Laboratories of Analytical Biology, National Museum of Natural History, Smithsonian Institution, Washington, D.C. 20013 United States of America; 7 Département de Biologie, École Normale Supérieure de Lyon, Université de Lyon, UCB Lyon1, 46 Allée d’Italie, Lyon, France; 8 Team Evolution of Vertebrate Dentition, Institute of Functional Genomics of Lyon, ENS de Lyon, CNRS UMR 5242, Université de UCB Lyon1, 46 allée d’Italie, Lyon, France; 9 School of Biological Sciences, The University of Queensland, Brisbane, 4072 Australia; 10 Institut de Recherche pour le Développement – UR 227 CoReUs, LABEX “CORAIL”, UPVD, 66000 Perpignan, France; 11 Department of Invertebrate Zoology, National Museum of Natural History, National Museum of Natural History, Smithsonian Institution, Washington, D.C. 20560-0163 United States of America; 12 UMR 248 MARBEC (IRD, Ifremer, Univ. Montpellier, CNRS), Station Ifremer de Sète, Av Jean Monnet, CS 30171, 34203 Sète cedex, France; 13 Institut Systématique, Évolution, Biodiversité (ISYEB), UMR 7205, CNRS, MNHN, UPMC, EPHE. Ecole Pratique des Hautes Etudes, Paris Sciences Lettres (PSL), 57 rue Cuvier, CP39, F-75005 Paris, France

**Keywords:** Genetic databases, DNA sequencing, Biodiversity, Taxonomy

## Abstract

The emergence of DNA barcoding and metabarcoding opened new ways to study biological diversity, however, the completion of DNA barcode libraries is fundamental for such approaches to succeed. This dataset is a DNA barcode reference library (fragment of Cytochrome Oxydase I gene) for 2,190 specimens representing at least 540 species of shore fishes collected over 10 years at 154 sites across the four volcanic archipelagos of French Polynesia; the Austral, Gambier, Marquesas and Society Islands, a 5,000,000 km^2^ area. At present, 65% of the known shore fish species of these archipelagoes possess a DNA barcode associated with preserved, photographed, tissue sampled and cataloged specimens, and extensive collection locality data. This dataset represents one of the most comprehensive DNA barcoding efforts for a vertebrate fauna to date. Considering the challenges associated with the conservation of coral reef fishes and the difficulties of accurately identifying species using morphological characters, this publicly available library is expected to be helpful for both authorities and academics in various fields.

## Abstract (summary)

**Table:** 

Design Type(s)	population data analysis objective • biodiversity assessment objective
Measurement Type(s)	fish
Technology Type(s)	taxonomic diversity assessment by targeted gene survey
Factor Type(s)	Species • geographic location
Sample Characteristic(s)	Actinopterygii • French Polynesia • ocean biome

Machine-accessible metadata file describing the reported data (ISA-Tab format)

## Background & Summary

DNA barcoding aims to identify individuals to the species level by using a short and standardized portion of a gene as a species tag^1^. This standardized procedure has revolutionized how biodiversity can be surveyed as the identification of a species then becomes independent of the level of taxonomic expertise of the collector^2^, the life stage of the species^3,4^ or the state of conservation of the specimen^5,6^. Due to its large spectrum of potential applications, DNA barcoding has been employed in a large array of scientific fields such as taxonomy^7^, biogeography, biodiversity inventories^8^ and ecology^9^; but see Hubert and Hanner for a review^10^. In the genomic era, this approach has been successfully applied to the simultaneous identification of multiple samples (*i.e*. the metabarcoding approach), extending its applications to surveys of whole ecological communities^11^, but also monitoring species diet^12,13^, identifying the presence of specific species in a region^14^, or studying changes in the community through time by sampling environmental DNA^15,16^.

By design, DNA barcoding has proved to be fast and accurate, but its accuracy is highly dependent on the completeness of DNA barcode reference libraries. These libraries turn surveys of Operational Taxonomic Units (OTUs) into species surveys through the assignment of species names to OTUs^17,18^, hence giving meaning to data for ecologists, evolutionary biologists and stakeholders. Taxonomists increasingly provide DNA barcodes of new species they are describing; but thousands of species of shore fishes still lack this diagnostic molecular marker.

In the South Pacific, an early initiative led by the CRIOBE Laboratory was successfully carried out for French Polynesian coral reef fishes at the scale of one island, Moorea (Society Island)^19^. The fish fauna of Moorea’s waters is one of the best known of the region given the historical operation of research laboratories and long term surveys^20,21^. The Moorea project revealed a high level of cryptic diversity in Moorea’s fishes^19^ and motivated the CRIOBE Laboratory to extend this biodiversity survey of shore fishes to the remaining islands of French Polynesia. French Polynesia (FP) is a 5,000,000 km^2^ region located between 7° and 27° South Latitude that constitutes a priority area for conducting a barcoding survey. This region is species rich due to its position at the junction of several biogeographic areas with varying levels of endemism. For example, the Marquesas Islands (northeastern FP) rank as the third highest region of endemism for coral reef fishes in the Indo-Pacific (13.7%^22^). The Austral Islands (southwestern FP) and Gambier Islands (southeastern FP) host numerous southern subtropical endemic species^23–25^. Finally, the Society Islands (western FP) possess the highest species richness (877 species) and the highest number of widespread species in French Polynesia^26^.

**Fig. 1:**
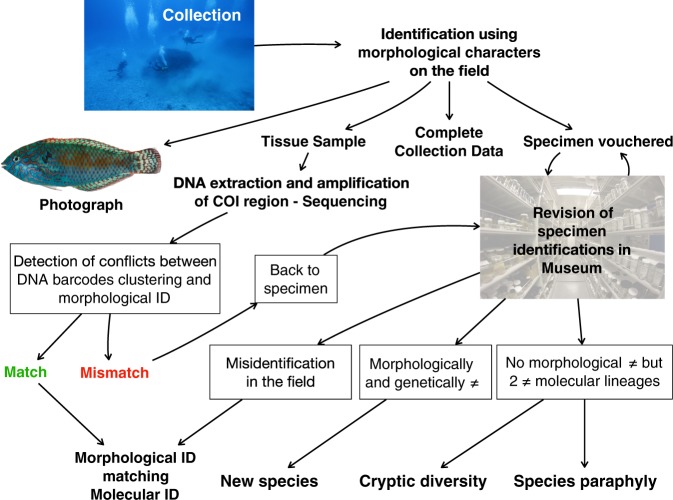
Overview of data generation. From collection of specimen to the validation of data generation.

Here, we present the result of a large-scale effort to DNA barcode the shore fishes in French Polynesia. Conducted between 2008 and 2014, a total of 154 sites were inventoried across these four archipelagoes. Islands of varying ages and topographies were visited ranging from low-lying atolls to high islands surrounded by a barrier reef, or solely fringing reefs. Furthermore, inventories were conducted across different habitats at each island (*i.e*. sand bank, coral reefs, rubble, rocky, etc.). In total, 2,190 specimens were identified, preserved, photographed, tissue sampled, DNA barcoded and cataloged with extensive metadata to build a library representing at least 540 species, 232 genera and 61 families of fishes (Fig. 1). Merged with previous sampling efforts at Moorea, a total of 3,131 specimens now possess a DNA barcode representing at least 645 nominal species for a coverage of approximately 65% of the known shore fish species diversity of these four archipelagoes. These biodiversity surveys have already resulted in the publication of updated species checklists^22,26^ and in the description of 17 new species^27–34^. This comprehensive library for French Polynesia shore fishes will certainly benefit a wide community of users with different interests, ranging from basic to applied science, and including fisheries management, functional ecology, taxonomy and conservation. Furthermore, many newly detected taxa for science are revealed here, along with complete collection data and DNA barcodes, which should facilitate their formal description as new species. While shedding new light on the species diversity of the Pacific region, this publicly available library is expected to fuel the development of DNA barcode libraries in the Pacific Ocean and to provide more accurate results for the growing number of studies using DNA metabarcoding in the Indo-West Pacific.

## Methods

### Sampling strategy

**Fig. 2:**
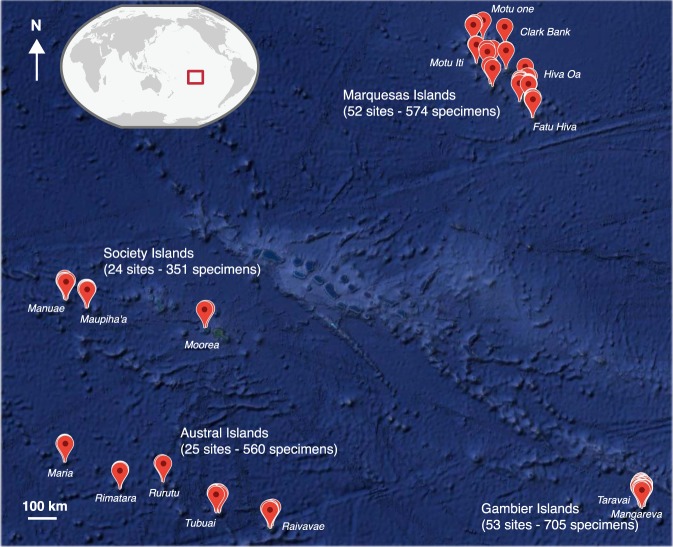
Sampling localities across French Polynesia. The number of sampling sites and the number of specimens collected are displayed for each archipelago. Several sampling localities may be represented by a single dot due to the geographic scale of French Polynesia. Map data: Google, DigitalGlobe.

**Fig. 3:**
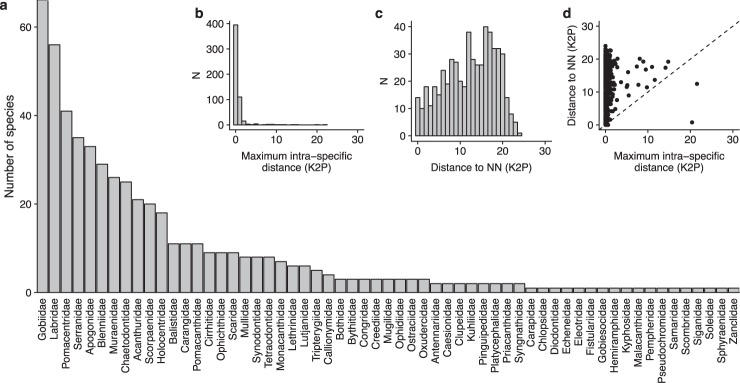
Species diversity and distribution of genetic distance across the DNA barcode library. (**a**) Species diversity by family for the four archipelagoes sampled; (**b**) Distribution of maximum intraspecific distances (K2P, percent); (**c**) Distribution of nearest neighbor distances (K2P, percent); (**d**) Relationship between maximum intraspecific and nearest neighbor distances. Points above the diagonal line indicate species with a barcode gap.

We explored a diversity of habitats across the four corners of French Polynesia with shallow and deep SCUBA dives (down to 50–55 m) for a total of 154 sampled sites (Fig. 2, Table 1). A total of 2,190 specimens, representing at least 540 species, 232 genera and 61 families (Fig. 3a) have been collected across four archipelagos representing the four corners of French Polynesia (FP), through six scientific expeditions: Marquesas Islands (1) in 2008 at Mohotani and (2) in 2011 at every island of the archipelago aboard the M.V. Braveheart (Clark Bank, Motu One, Hatutaa, Eiao, Motu Iti, Nuku-Hiva, Ua-Huka, Ua-Pou, Fatu-Huku, Hiva-Oa, Tahuata, Fatu-Hiva; 52 sites), (3) in 2010 at Gambier Islands aboard the M.V. Claymore (Mangareva, Taravai, Akamaru, and all along the barrier reef; 53 sites), (4) at Austral Islands in 2013 aboard the Golden Shadow (Raivavae, Tubuai, Rurutu, Rimatara, Maria Islands; 25 sites), (5) at westernmost atolls of the Society Islands in 2014 aboard the M.V. Braveheart (Manuae and Maupiha’a; 20 sites). A sixth scientific expedition took place on Moorea’s deep reefs in 2008 (Society Islands) as a small scale scientific expedition that included the exploration and sampling of some of the deep reefs of Moorea (53 to 56 m depth; 4 sites) (Fig. 2).

**Table 1:** Overview of the dataset.

BOLD project	Geographical location	No. of specimen collected	No. of species collected	Sampling effort (No. of sampling days/No. of sites)
AUSTR	Austral Islands	560	263	12/25
GAMBA	Gambier Islands	705	290	18/53
MARQ	Marquesas Islands	386	182	18/41
MOH	Marquesas Islands	190	107	5/11
MOOP	Society Islands	42	27	4/4
SCIL	Society Islands	309	213	8/20

Number of specimens and species collected for each scientific expedition. Sampling effort expressed in number of sampling days and number of sites.

### Specimen collection

Specimens were captured using rotenone (powdered root of the Derris plant) and spear guns while SCUBA diving. These complementary sampling methods^35^ allowed us to sample both the cryptic and small fish fauna as well as the larger specimens of species not susceptible to rotenone collecting. Four individuals per species were collected on average. Fishes were sorted and identified onboard to the species level using identification keys and taxonomic references^23,36^ and representative specimens of all species collected were photographed in a fish photo tank to capture fresh color patterns, labeled and tissue sampled for genetic analyses (fin clip or muscle biopsies preserved in 96% ethanol). The photographed/sampled voucher specimens were preserved in 10% formalin (3.7% formaldehyde solution) and later transferred into 75% ethanol for permanent archival storage. Preserved voucher specimens and tissues were deposited and cataloged into the fish collection at the Museum Support Center, National Museum of Natural History, Smithsonian Institution, Suitland, Maryland, USA. Nomenclature follows Randall^23^ and we followed recent taxonomic changes using the California Academy of Sciences Online Eschmeyer’s Catalog of Fishes^37^.

### DNA barcode sequencing

We extracted whole genomic DNA using QIAxtractor (QIAGEN, Crawley) and Autogen AutoGenPrep 965 according to manufacturer’s protocols. A 655 bp fragment of the cytochrome oxidase I gene (COI) was amplified using Fish COI primers FISHCOILBC (TCAACYAATCAYAAAGATATYGGCAC) and FISHCOIHBC (ACTTCYGGGTGRCCRAARAATCA) and Polymerase Chain Reaction (PCR) and Sanger sequencing protocols as in Weigt *et al*.^38^. PCR products were Sanger sequenced bidirectionally and run on an ABI3730XL in the Laboratories of Analytical Biology (National Museum of Natural History, Smithsonian Institution). Sequences were edited using Sequencher 5.4 (Gene Codes) and aligned with Clustal W as implemented in Barcode Of Life Datasystem (BOLD, http://www.boldsystems.org). Alignments were unambiguous with no indels or frameshift mutations. A total of 2,190 DNA barcodes have been generated.

### Specimen identification

All morphological identifications were revised as needed after the specimens were deposited in the archival specimen collection to confirm initial identifications made in the field. Specimens of specific groups like Antennaridae, Bythitidae, Chlopsidae or Muraenidae were revised by additional taxonomist specialists (David Smith, John McCosker, Leslie W. Knapp, Werner Schwarzhans). After the morphological identification, we used the Taxon-ID Tree tool and Barcode Index Numbers (BIN) discordance tools as implemented in the Sequence Analysis module of BOLD to check every identification using the DNA barcodes generated. The Taxon-ID tool consists of the construction of a neighbor-joining (NJ) tree using K2P (Kimura 2 Parameter) distances by BOLD to provide a graphic representation of the species divergence^39^. The BIN discordance tool uses the Refined Single Linkage algorithm (RESL^40^) to provide a total number of OTUs.

## Data Records

This library is composed of three main components: (1) voucher specimens archived in the national fish collection at the Smithsonian Institution (Washington, DC), which were photographed in the field, (2) complete collection data associated with each voucher specimen, and (3) DNA barcodes (Fig. 1).

All photographs, voucher collection numbers, DNA barcodes and collection data are publicly available in BOLD^41^ in the Container INDOF “Fish of French Polynesia” or by scientific expedition (“AUSTR”, “GAMBA”, “MARQ”, “MOH”, “MOOP” and “SCILL”) and in Figshare^42^. DNA barcodes have also been made available in GenBank, and have accessions KC567661^43^ to KC567663^44^, KC684990^45^, KC684991^46^, KU905709^47^ to KU905727^48^, KY570698^49^, KY570703^50^ to KY570705^51^, KY570708^52^, KY683549^53^, MH707846^54^ to MH707881^55^, MK566774^56^ to MK567153^57^, MK656969^58^ to MK658713^59^ and this database is accessible through the CRIOBE portal (http://fishbardb.criobe.pf).

The library fulfills the BARCODE data standard^60,61^ which requires: 1) Species name, 2) Voucher data, 3) Collection data, 4) Identifier of the specimen, 5) COI sequence of at least 500 bp, 6) PCR primers used to generate the amplicon, 7) Trace files. In BOLD, each record in a project represents a voucher specimen with its photographs, voucher collection numbers, associated sequences and extensive collection data related to (1) the Voucher: Sample ID, Field ID, Museum ID, Institution Storing; (2) the Taxonomy: Phylum, Class, Order, Family, Subfamily, Genus, species, Identifier, Identifier E-mail, Taxonomy Notes; (3) Specimen Details: Sex, Reproduction, Life Stage, FAO Zone, Notes such as sizes of the specimens, Voucher Status, and (4) Collection Data: Collectors, Collection Date, Continent, Country/Ocean*, State/Province, Region, Sector, Exact Site, GPS Coordinates, Elevation, Depth, Depth Precision, GPS Source, and Collection Notes^42^.

## Technical Validation

To test the robustness of our library, we first computed the distribution of the interspecific and intraspecific variability for all the described species (Fig. 3b–d). We found that there is little to no overlap in the distribution of divergence within and between species for the vast majority of the species identified morphologically (mean intra-specific divergence 0.66, min: 0.00, max: 21.56; mean inter-specific divergence 12.28, min: 0.00, max: 24.01). The RESL algorithm identified more BINs (617) than nominal species identified morphologically (540). The morphological reexamination of specimens in light of these results suggest that 65 taxa could be new species for science awaiting a formal description (Online-only Table 1) as they are morphologically distinguishable from other species and possess unique BIN numbers. Taxonomic paraphyly (*i.e*. potentially cryptic species) has been found for 18 additional species (Table 2) as they are divided in 37 different BINs, while no morphological character has been found so far to distinguish them. Finally, mixed genealogies between sister-species were observed for 17 species (Table 3), mostly between some of the Marquesan endemics and their closest relatives that are not currently observed in the Marquesas Islands. Considering the maternal inheritance of the mitochondrial genes and the very shallow genealogies involved (maximum K2P genetic distances lower than 2%), both incomplete lineage sorting and past introgressive hybridization might be responsible of the mixing of species genealogies in those 17 cases. In summary, 94% of the BINs match species identified using morphological characters, meaning that it was possible to successfully identify a species using DNA barcodes in 94% of the cases.

**Table 2:** Potential cryptic species.

BINs	Taxa	No. of specimens
BOLD:AAF8427	*Apogon crassiceps*	2
BOLD:ABW7007	*Apogon crassiceps*	4
BOLD:ACE7901	*Apogon crassiceps*	1
BOLD:ACX1964	*Apogon doryssa*	1
BOLD:ABW8494	*Apogon doryssa*	2
BOLD:AAF5636	*Aporops bilinearis*	1
BOLD:AAF5637	*Aporops bilinearis*	4
BOLD:AAD2580	*Centropyge flavissima*	2
BOLD:AAD9019	*Centropyge flavissima*	6
BOLD:ACD1956	*Fusigobius duospilus*	5
BOLD:AAD1050	*Fusigobius duospilus*	1
BOLD:AAA6306	*Gnatholepis cauerensis*	9
BOLD:AAC6155	*Gnatholepis cauerensis*	5
BOLD:ACC5235	*Gymnothorax melatremus*	3
BOLD:AAC8364	*Gymnothorax melatremus*	5
BOLD:AAF0704	*Leiuranus semicinctus*	3
BOLD:AAL6561	*Leiuranus semicinctus*	2
BOLD:ACD1820	*Myrophis microchir*	1
BOLD:AAE0976	*Myrophis microchir*	2
BOLD:AAB3862	*Parupeneus multifasciatus*	6
BOLD:ACD1989	*Parupeneus multifasciatus*	3
BOLD:ACD1988	*Priolepis triops*	3
BOLD:AAX7961	*Priolepis triops*	1
BOLD:AAB4082	*Pristiapogon kallopterus*	1
BOLD:ABZ7996	*Pristiapogon kallopterus*	7
BOLD:ACC5180	*Pseudocheilinus octotaenia*	10
BOLD:AAD3038	*Pseudocheilinus octotaenia*	9
BOLD:AAB4821	*Pterocaesio tile*	4
BOLD:ACK9118	*Pterocaesio tile*	1
BOLD:ACP9778	*Scolecenchelys gymnota*	1
BOLD:AAJ8783	*Scolecenchelys gymnota*	2
BOLD:AAC7090	*Stegastes fasciolatus*	11
BOLD:ABZ0285	*Stegastes fasciolatus*	2
BOLD:ACC5053	*Uropterygius kamar*	1
BOLD:ACC5109	*Uropterygius kamar*	1
BOLD:ACD1642	*Uropterygius macrocephalus*	1
BOLD:AAU1965	*Uropterygius macrocephalus*	2

Species with number of specimens collected displaying taxonomic paraphyly most likely representing undescribed cryptic species. Sample ID includes sampling location (AUST: Austral Islands, GAMB: Gambier Islands, MARQ and MOH: Marquesas Islands, SCIL and MOOP: Society Islands).

**Table 3:** Species displaying either incomplete lineage sorting or shallow inter-species divergence.

Family	Species	Mean Intra-Sp	Max Intra-Sp	Nearest Neighbour	Nearest Species	Distance to NN
Acanthuridae	*Acanthurus reversus*	0.08	0.15	AUSTR453-13	*Acanthurus olivaceus*	0
Holocentridae	*Myripristis earlei*	0.28	0.62	SCILL065-15	*Myripristis berndti*	0
Monacanthidae	*Pervagor marginalis*	0.36	0.62	SCILL083-15	*Pervagor aspricaudus*	0
Tetraodontidae	*Canthigaster criobe*	0	0	MOH030-16	*Canthigaster janthinoptera*	0
Mullidae	*Mulloidichthys mimicus*	0.52	0.52	AUSTR089-13	*Mulloidichthys vanicolensis*	0.17
Pomacentridae	*Chromis abrupta*	0	0	SCILL209-15	*Chromis margaritifer*	0.31
Labridae	*Coris marquesensis*	0	0	SCILL040-15	*Coris gaimard*	0.46
Apogonidae	*Ostorhinchus relativus*	N/A	0	SCILL142-15	*Ostorhinchus angustatus*	0.93
Tetraodontidae	*Canthigaster rapaensis*	0.21	0.31	MARQ456-12	*Canthigaster marquesensis*	1.1
Pomacentridae	*Abudefduf conformis*	0.15	0.15	GAMBA844-12	*Abudefduf sexfasciatus*	1.24
Monacanthidae	*Cantherhines nukuhiva*	0.15	0.31	GAMBA711-12	*Cantherhines sandwichiensis*	1.4
Pomacentridae	*Plectroglyphidodon sagmarius*	0.08	0.15	AUSTR222-13	*Plectroglyphidodon imparipennis*	1.56
Holocentridae	*Sargocentron caudimaculatum*	0.68	1.1	SCILL104-15	*Sargocentron tiere*	1.57
Acanthuridae	*Zebrasoma rostratum*	0	0	AUSTR376-13	*Zebrasoma scopas*	1.72
Apogonidae	*Apogon marquesensis*	0.23	0.31	GAMBA657-12	*Apogon susanae*	1.88
Chaetodontidae	*Chaetodon flavirostris*	0.08	0.15	SCILL269-15	*Chaetodon lunula*	1.88
Chaetodontidae	*Chaetodon lunula*	0.1	0.15	GAMBA555-12	*Chaetodon flavirostris*	1.88

Mean and Maximum intra-Species distances (Mean Intra-Sp and Max Intra-Sp), and Kimura 2 Parameter distances from the Nearest Neighbour (NN).

## Usage Notes

This Barcode release dataset is freely available to use in barcoding or metabarcoding surveys for specimen identification. Several approaches can be considered:

1. directly downloading the sequences in fasta format, and working offline by merging this dataset with an ongoing barcoding project;
2. working online, through the BOLD website (registration is free), and merging the Container INDOF “Fish of French Polynesia” or parts of the scientific expeditions (Table 1) with an ongoing BOLD project;
3. through online identification tools, as data are indexed in both BOLD and Genbank databases. This library will be considered when any queries of molecular identification will be made through the identification engine of BOLD (http://www.boldsystems.org/index.php/IDS-OpenIdEngine) or the standard nucleotide Basic Local Alignment Search Tool (BLAST, https://blast.ncbi.nlm.nih.gov/). In the same manner, this dataset should also be indexed in the MIDORI database^62,63^. Composed of both endemic and widespread species, this library is expected to benefit a large community from academics to authorities who use molecular data to monitor and survey biodiversity.

### ISA-Tab metadata file

Download metadata file

## Online-only Table

**Online-only Table 1:** Potential new species detected in the dataset.

Sample ID	Family	Genus	Species	No. of specimens
AUST-419	Antennariidae	*Antennatus*	sp1	1
AUST-570, GAM-355	Antennariidae	*Antennatus*	sp2	2
MARQ-022, MOH-087	Antennariidae	*Antennatus*	sp3	2
SCIL-293	Antennariidae	*Antennatus*	sp4	1
MARQ-105, MARQ-106, MARQ-107	Apogonidae	*Fowleria*	sp	3
MARQ-380, MARQ-381, MOH-068	Apogonidae	*Gymnapogon*	sp	3
AUST-142, AUST-143	Apogonidae	*Pseudamiops*	sp	2
AUST-470, AUST-471	Apogonidae	*Siphamia*	sp	2
MARQ-177, MARQ-208, MOH-040	Blenniidae	*Blenniella*	sp	3
AUST-242, GAM-791, GAM-792, MARQ-139, MARQ-140	Blenniidae	*Cirripectes*	*sp*	5
GAM-278, GAM-279, SCIL-017, SCIL-021, SCIL-058, SCIL-288, SCIL-322	Blenniidae	*Enchelyurus*	*sp*	7
AUST-407, AUST-408, AUST-409	Blenniidae	*Entomacrodus*	sp	3
MARQ-184, MARQ-187, MARQ-378, MARQ-379	Blenniidae	*Rhabdoblennius*	sp	4
MOOP-028	Chlopsidae	*Kaupichthys*	sp	1
AUST-600	Congridae	*Ariosoma*	sp1	1
MARQ-318	Congridae	*Ariosoma*	sp2	1
MARQ-314, MARQ-315, MARQ-316	Congridae	*Gnathophis*	sp	3
MARQ-397, MOH-062	Creediidae	*Chalixodytes*	sp	1
AUST-427, AUST-538, AUST-539	Creediidae	*Crystallodytes*	*sp*	3
AUST-413	Gobiesocidae	*NA*	sp	1
AUST-159, AUST-305	Gobiesocidae	*Pherallodus*	sp1	2
AUST-532, AUST-533, AUST-534	Gobiesocidae	*Propherallodus*	sp	3
AUST-082	Gobiidae	*Bryaninops*	sp	1
GAM-374, GAM-375	Gobiidae	*Cabillus*	sp	2
MARQ-430, MOH-129, MOH-211	Gobiidae	*Callogobius*	sp	3
AUST-303, GAM-379	Gobiidae	*Eviota*	sp1	1
GAM-697	Gobiidae	*Eviota*	sp2	1
SCIL-038	Gobiidae	*Eviota*	sp3	1
AUST-346, AUST-347	Gobiidae	*Gobiodon*	sp	2
MARQ-097, MARQ-098	Gobiidae	*Gobiodon*	sp	2
SCIL-240	Gobiidae	*Gobiodon*	sp	1
AUST-566, AUST-567, AUST-568, GAM-364	Gobiidae	*Paragobiodon*	sp	4
MARQ-363, MARQ-364, MARQ-365	Gobiidae	*Pleurosicya*	sp1	3
MOOP-007	Gobiidae	*Pleurosicya*	sp2	1
SCIL-237	Gobiidae	*Priolepis*	sp	1
AUST-032	Gobiidae	*Silhouettea*	sp	1
MOOP-047	Gobiidae	*Sueviota*	sp	1
AUST-446	Gobiidae	*Trimma*	sp1	1
MARQ-435, MARQ-436	Gobiidae	*Trimma*	sp2	2
GAM-32	Gobiidae	*Trimmatom*	sp1	1
GAM-33	Gobiidae	*Trimmatom*	sp2	1
MOOP-002	Gobiidae	*Trimmatom*	sp3	1
AUST-423, AUST-424, AUST-425, AUST-544	Isonidae	*Iso*	sp1	4
GAM-521, GAM-522	Isonidae	*Iso*	sp2	2
MOH-079	Lethrinidae	*Gymnocranius*	sp	1
AUST-383, GAM-176, GAM-177, GAM-178, MOH-200, SCIL-299	Moringuidae	*Moringua*	sp1	6
SCIL-300	Moringuidae	*Moringua*	sp2	1
MARQ-505	Muraenidae	*Gymnothorax*	sp1	1
SCIL-335	Muraenidae	*Gymnothorax*	sp2	1
AUST-573, GAM-709	Ophidiidae	*Brotula*	sp	2
AUST-297, AUST-298, AUST-299, AUST-300, GAM-761	Pempheridae	*Pempheris*	sp1	5
MARQ-063, MARQ-165, MARQ-166, MARQ-167, MARQ-276, MARQ-382, MOH-178, MOH-179	Pempheridae	*Pempheris*	sp2	8
AUST-308, AUST-309, AUST-310, AUST-311	Pomacentridae	*Stegastes*	sp	4
MOOP-018, MOOP-019, MOOP-034, MOOP-035	Pseudochromidae	*Lubbockichthys*	sp	4
GAM-599, GAM-600	Scorpaenidae	*Scorpaenodes*	sp1	2
MOH-137, MOH-151	Scorpaenidae	*Scorpaenodes*	sp2	2
GAM-56, GAM-569, GAM-574, GAM-58	Scorpaenidae	*Sebastapistes*	sp1	4
MARQ-328, SCIL-114	Scorpaenidae	*Sebastapistes*	sp2	1
MARQ-046, MOH-027	Syngnathidae	*Doryrhamphus*	sp	1
MARQ-321	Synodontidae	*Synodus*	sp	1
AUST-011, AUST-048	Tripterygiidae	*Enneapterygius*	sp1	1
AUST-360, AUST-057, AUST-058, AUST-059	Tripterygiidae	*Enneapterygius*	sp2	4
GAM-68, GAM-69, GAM-123, GAM-124, GAM-125	Tripterygiidae	*Enneapterygius*	sp3	3
GAM-002	Tripterygiidae	*Enneapterygius*	sp4	1
SCIL-112, SCIL-133, SCIL-156	Tripterygiidae	*Enneapterygius*	sp5	3

Specimens which were identified only to the genus level and which represent potentially new species waiting to be described. Number of specimens included in each Barcode Index Number (BIN).
